# *Mycobacterium leprae* in Armadillo Tissues from Museum Collections, United States

**DOI:** 10.3201/eid2903.221636

**Published:** 2023-03

**Authors:** Daniel Romero-Alvarez, Daniel Garzon-Chavez, Mary Jackson, Charlotte Avanzi, A. Townsend Peterson

**Affiliations:** Universidad de las Américas, Quito, Ecuador (D. Romero-Alvarez);; University of Kansas, Lawrence, Kansas, USA (D. Romero-Alvarez, A.T. Peterson);; Universidad San Francisco de Quito, Quito, Ecuador (D. Garzon-Chavez);; Colorado State University, Fort Collins, Colorado, USA (M. Jackson, C. Avanzi)

**Keywords:** *Mycobacterium leprae*, armadillo, Hansen disease, leprosy, PCR, tuberculosis and other mycobacteria, molecular techniques, *Mycobacterium lepromatosis*, museum collections, United States, Bolivia, Paraguay, *Suggested citation for this article*: Romero-Alvarez D, Garzon-Chavez D, Jackson M, Avanzi C, Peterson AT. *Mycobacterium leprae* in armadillo tissues from museum collections, United States. Emerg Infect Dis. 2023 Mar [*date cited*]. https://doi.org/10.3201/eid2903.221636

## Abstract

We examined armadillos from museum collections in the United States using molecular assays to detect leprosy-causing bacilli. We found *Mycobacterium leprae* bacilli in samples from the United States, Bolivia, and Paraguay; prevalence was 14.8% in nine-banded armadillos. US isolates belonged to subtype 3I-2, suggesting long-term circulation of this genotype.

Hansen disease (leprosy) is an ancient pathology caused by 2 slow-growing intracellular bacilli, *Mycobacterium leprae* and *M. lepromatosis* (*1*). Both pathogens have the ability to damage peripheral nerves of hosts, producing a broad spectrum of clinical outcomes. Routes of disease transmission have been hypothesized for >100 years but are still actively debated (*2*); traditionally, human-to-human transmission has been considered the dominant route of infection. Evidence incriminates *M. leprae* as a zoonotic pathogen; the nine-banded armadillo (*Dasypus novemcinctus*) is its main wildlife reservoir in the southern United States (*2*). *M. leprae* also has been found in *D. novemcinctus* armadillos outside the United States (e.g., in Brazil), in the six-banded armadillo (*Euphractus sexcinctus*), and in nonhuman primates including chimpanzees, macaques, and sooty mangabeys (*2*). In addition, *M. leprae* and *M. lepromatosis* have been reported in red squirrels (*Sciurus vulgaris*) in the British Isles (*3*). Those data strongly suggest broad zoonotic transmission dynamics for both bacilli.

Natural history collections represent a neglected resource for biomedical research despite their known utility (*4*). We examined armadillo (family Dasypodidae) tissues deposited in museum collections across the United States to identify *M. leprae* and *M. lepromatosis* across space and time. We report presence of *M. leprae* in armadillo tissue samples from endemic and nonendemic areas of the Americas, suggesting that public health policy should contemplate zoonotic leprosy transmission routes carefully.

## The Study

**Figure 1:**
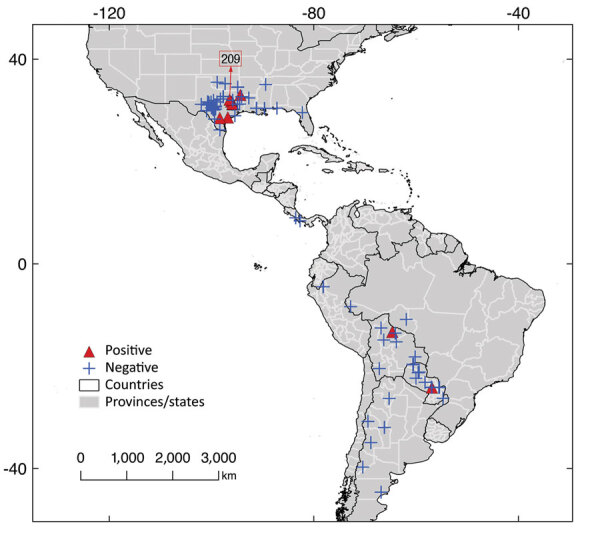
Geographic origin of samples analyzed in study of *Mycobacterium leprae* in armadillo tissue samples from US museums (n = 8 countries). We obtained coordinates from the tissue metadata or georeferenced them manually by using Google Earth (https://earth.google.com). Of the 2 samples suitable for whole-genome sequencing, 1, USA-am-109, lacked spatial detail from which to obtain coordinates and is not included on the map, along with 4 additional samples. The other sample that was sequenced, USA-am-209, is indicated with an arrow and the number in a red square.

We assembled a database of museum armadillo tissue samples using the biodiversity information portals VertNet (http://portal.vertnet.org) and Arctos (https://arctos.database.museum/home.cfm), queried during December 2018–April 2019. Ten US museums included armadillo samples in their datasets. The samples were collected during 1974–2017 (Appendix 1 Figure 4) from 8 countries across the Americas; 68.6% (n = 109) came from the United States (Table 1; Figure 1). Each museum contributed ≈1 mm^3^ of armadillo tissue (Appendix 1; Appendix 2). The 159 samples processed corresponded to 10 armadillo species; *D. novemcinctus*, the nine-banded armadillo, was the most common (n = 122 [76.7%]). Most samples were liver tissues (n = 66 [41.5%]), followed by muscle (n = 37 [23.3%]) and spleen (n = 31 [19.5%]) (Table 1). The specimens were frozen or preserved in 10% dimethyl sulfoxide or 70%, 90%, or 95% ethanol; most were either frozen (n = 77 [48.4%]) or in 95% ethanol (n = 55 [34.6%]) (Table 1). 

**Table 1:** Characteristics of armadillo tissues from US museum collections examined for Mycobacterium leprae and M. lepromatosis*

Category	No. (%) animals	No. (%) positive for *M. leprae*
Species		
* Dasypus novemcinctus *	122 (76.7)	18 (100)
* Tolypeutes matacus*	20 (12.6)	0
* Cabassous unicinctus*	5 (3.1)	0
* Chaetophractus vellerosus*	3 (1.9)	0
* Zaedypus pichiy*	3 (1.9)	0
* Chaetophractus villosus*	2 (1.3)	0
* Cabassous tatouay*	1 (0.6)	0
* Chaetophractus* sp.	1 (0.6)	0
* Euphractus sexcinctus*	1 (0.6)	0
* Priodontes maximus*	1 (0.6)	0
Total	159 (100)	18 (100)
Sex		
M	72 (45.3)	4 (22.2)
F	71 (44.7)	12 (66.7)
Unknown†	16 (10.1)	0
Total	159 (100)	18 (100)
Tissues tested		
Liver	66 (41.5)	2 (11.1)
Muscle	37 (23.3)	13 (72.2)
Spleen	31 (19.5)	3 (16.7)
Unknown	16 (10.1)	0
Lysate	4 (2.5)	0
Heart and kidney	2 (1.3)	0
Kidney	2 (1.3)	0
Heart	1 (0.6)	0
Total	159 (100)	18 (100)
Preservation method		
Frozen	77 (48.4)	4 (22.2)
Ethanol 95%	55 (34.6)	13 (72.2)
Ethanol 70%	17 (10.7)	0
DMSO	9 (5.7)	1 (5.6)
Ethanol 90%	1 (0.6)	0
Total	159 (100)	18 (100)
DNA concentration, ng/µL		
Mean	19	15.63
SD	27.3	12.2
Range	0.0041–218	0.0041–43
Country of origin		
United States	109 (68.6)	16 (88.9)
Paraguay	24 (15.1)	1 (5.6)
Argentina	10 (6.3)	0
Bolivia	7(4.4)	1 (5.6)
Peru	3 (1.9)	0
Brazil	2 (1.3)	0
Unknown	2 (1.3)	0
Costa Rica	1 (0.6)	0
Panama	1 (0.6)	0
Total	159 (100)	18 (100)

*All samples tested negative for *M. lepromatosis*. DMSO, dimethyl sulfoxide. †Unknown indicates no data were available.

We processed tissues using an in-house DNA extraction method based on magnetic beads (Appendix 1). We applied standardized PCR protocols using specific primers to detect *M. leprae* and *M. lepromatosis* (*5*,*6*). For *M. leprae*, we implemented typification and subtypification as described previously (*7*). We performed quantitative real-time PCR (qPCR) on all samples for which genotyping was successful as a proxy of *M. leprae* DNA quantity with cycle threshold (Ct) <26 as a threshold for whole-genome sequencing. We multiplexed and sequenced libraries on an Illumina NextSeq 500 instrument (https://www.illumina.com) (Appendix 1).

**Figure 2:**
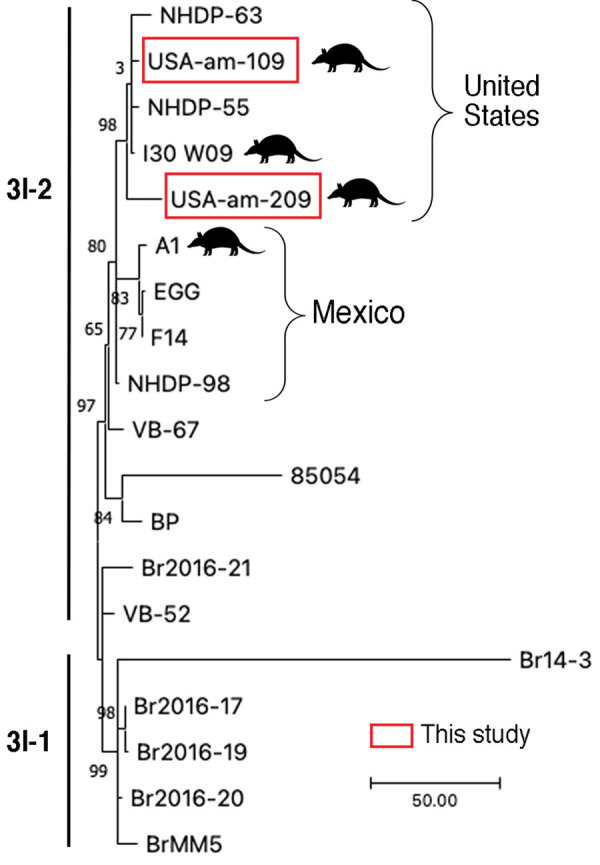
Comparative genomics of the *Mycobacterium leprae* sequenced this study from armadillo tissues from US museums and those from humans and armadillos from the United States and Mexico. Samples subjected to whole-genome sequencing, USA-am-109 and USA-am-209, clustered among genomes from humans and armadillos from the United States (branch 3I). The tree represents a zoom into the *M. leprae* genotypes 3I-1 and 3I-2 from a maximum-parsimony tree of 302 *M. leprae* genomes rooted with *M. lepromatosis* as outgroup. The tree was built in MEGA version 11 software (https://www.megasoftware.net). Support values were obtained by bootstrapping 500 replicates. Scale bar indicates number of nucleotide substitutions.

We found *M. leprae* in 18/159 (11.3%) samples. All positives were in *D. novemcinctus* armadillos, for prevalence in that species of 14.8% (n = 18/122). We detected positive results mainly in muscle tissue (n = 13/18 [72.2%]) and in 95% ethanol–preserved specimens (n = 13/18 [72.2%]) (Tables 1, 2). *M. lepromatosis* was not detected in the tissues examined. PCR subtyping was successful in 5/18 (27.8%) positive samples; 4 belonged to subtype 3I, as expected for armadillos from Texas, USA (*8*) (Table 2). The remaining sample was characterized only to type (3 or 4), because we found low amounts of *M. leprae* DNA (Table 2). After RLEP qPCR, 2 samples had a Ct<26 (i.e., 109 and 209) and were suitable for whole-genome sequencing. The genomes of *M. leprae* National Center for Biotechnology Information BioSample no. SAMN31421191 (https://www.ncbi.nlm.nih.gov/biosample) had coverage of 18.2× and of BioSample SAMN31421192, 4.9× (Appendix 1). Phylogenetic analysis showed that both *M. leprae* strains belonged to genotype 3I-2 (*8*,*9*). The 2 *M. leprae* genomes clustered specifically with other isolates previously identified in armadillos (i.e., I-30) and humans (i.e., NHDP-55 and NHDP-63) from the United States (Figure 2). Isolate 109 harbored 3 specific single-nucleotide polymorphisms, including 1 missense mutation in *argD* (i.e., C1691069T; Arg61Cys), encoding a probable acetylornithine aminotransferase. Sequence data are available from the National Center for Biotechnology Information Sequence Read Archive under accession no. PRJNA893376.

**Table 2:** Characteristics of armadillo tissue samples from US museums identified as positive by standard PCR for Mycobacterium leprae*

Voucher/tissue no.	Sample ID	Tissue type	Preservation (%)	DNA con.	Country	State	Sex	Year	Type	Subtype	Ct
YPM 16952	63	Muscle	Ethanol (95)	20	USA	Texas	F	2014	ND	ND	ND
YPM 15982	66	Muscle	Ethanol (95)	13	USA	Texas	F	2015	ND	ND	ND
YPM 15294	80	Muscle	Ethanol (95)	2.89	USA	Texas	F	2013	3	3I	34.41
YPM 16954	95	Muscle	Ethanol (95)	11	USA	Texas	M	2014	ND	ND	ND
YPM 15295	97	Muscle	Ethanol (95)	14	USA	Texas	F	2013	ND	ND	ND
YPM 15292	99	Muscle	Ethanol (95)	5.7	USA	Texas	F	2013	ND	ND	ND
YPM 15296	103	Muscle	Ethanol (95)	8.9	USA	Texas	F	2013	ND	ND	ND
YPM 15293	105	Muscle	Ethanol (95)	4.76	USA	Texas	M	2013	ND	ND	ND
**YPM 14944**	**109**	**Muscle**	**Ethanol (95)**	**9.3**	**USA**	**Texas**	**NA**	**2014**	**3**	**3I**	**23.15**
YPM 15315	110	Muscle	Ethanol (95)	0.0041	USA	Texas	F	2013	ND	ND	ND
YPM 15298	111	Muscle	Ethanol (95)	27	USA	Texas	F	2013	ND	ND	ND
YPM 15299	115	Muscle	Ethanol (95)	43	USA	Texas	F	2012	ND	ND	ND
UAM 46589	118	Liver	DMSO	11	Paraguay	Canindeyu	F	1996	ND	ND	ND
MSB 140243	138	Liver	Ethanol (95)	37	Bolivia	Beni	NA	1993	ND	ND	ND
TTU 75235	158	Spleen	Frozen	19	USA	Texas	F	1996	3 or 4	ND	35.12
TTU 82457	194	Muscle	Frozen	3.82	USA	Texas	M	2000	3	3I	31.58
**TTU 75360**	**209**	**Spleen**	**Frozen**	**20**	**USA**	**Texas**	**F**	**1996**	**3**	**3I**	**25.83**
TTU 80673	212	Spleen	Frozen	31	USA	Texas	M	1999	ND	ND	ND

*We identified a total of 18 *M. leprae*-positive samples. Bold text indicates samples suitable for whole-genome sequencing (n = 2). Samples negative for subtyping were determined unsuitable for whole-genome sequencing. Ct determined by quantitative PCR. Ct, cycle threshold; DNA con., concentration of total DNA per sample; NA, no data available; ND, not determined

## Conclusions

We identified *M. leprae* in *D. novemcinctus* armadillos only; prevalence was 14.8%. Positive samples were mainly detected from muscle and from ethanol-preserved specimens (Table 1). Infected armadillos were found in the United States, Paraguay, and Bolivia. *M. leprae* has not been reported in other wildlife in Paraguay or Bolivia. In our study, tissues from Paraguay were collected in 1996 and from Bolivia in 1993 (Table 2). Hansen disease is prevalent in humans in both countries (*10*); presence of infected armadillos should prompt research to explore their role as a potential zoonotic source of leprosy (*2*). In Bolivia, a previous survey of *D. novemcinctus* and *T. matacus* armadillos conducted during 1999–2001 found 0 positive animals (*2*). We found 7 *M. leprae*–negative armadillo tissues in the United States: 1 from Florida in 1974 and 6 from Texas collected during 1982–1990 (Appendix 2). No evidence for *M. leprae* was reported in Florida before 2009 (*8*). In Texas, although immunologic detection studies suggested the presence of *M. leprae* in armadillos before the 2000s, evidence was restricted to 1 area (*2*). Thus, our molecular identification of *M. leprae* in Texas armadillos from 1996, 1999, and 2000 are novel records (Table 2; Figure 1).

*M. lepromatosis* has been reported in multiple countries of the Americas, including the United States, Mexico, and Colombia, but as of 2022, only in humans (*11*,*12*). Although this species has been detected in *Sciurus vulgaris* squirrels in the British Isles, broader surveillance in rodents across Europe and Mexico identified 0 positive samples (*13*). From our dataset we obtained only negative results. *M. lepromatosis* is seldom screened as a Hansen disease–causing pathogen because of lack of awareness, which has impeded understanding of its incidence. Thus, in countries endemic for Hansen disease, *M. lepromatosis* should be also screened systematically in humans and potential animal reservoirs.

We were able to identify *M. leprae* subtypes in 4 armadillos (Table 2) and to sequence 2 entire genomes. Those 2 strains clustered with armadillo and human isolates from the United States, all belonging to subtype 3I-2, on branch 3 of the genetic tree (Figure 2). Of interest, our isolates differed by several nonsynonymous sites from those isolated previously. Our findings corroborate that several strains of *M. leprae* are circulating in armadillo populations in the southern United States (*8*,*9*,*14*). As predicted (*15*), our data also confirm that the strains circulating in armadillos today are close to those infecting animals >30 years ago, highlighting the promise of using preserved animal tissues to study epizootic dynamics of leprosy and other diseases.

Information on pathogen biodiversity in wildlife is much needed. We suggest that specimens in natural museums can play a role in infectious disease monitoring; our study relied on the global museum initiative and the large digital repositories of relevant specimen data in the United States. Protocols for using museum repositories for infectious disease research are still in development (*4*); parameters to optimal pathogen identification should be explored for *M. leprae* and other pathogens. We recognize that no single best way to study the diversity of pathogens exists; any approach should consider the specific nuances of each zoonotic system.

Appendix 1 Additional methods used in study of *Mycobacterium leprae* in armadillos.

Appendix 2 Details of the 159 specimens processed in study of *Mycobacterium leprae* in armadillos.

## References

[1] Singh P, Benjak A, Schuenemann VJ, Herbig A, Avanzi C, Busso P, et al. Insight into the evolution and origin of leprosy bacilli from the genome sequence of *Mycobacterium lepromatosis.* Proc Natl Acad Sci U S A. 2015;112:4459–64. 10.1073/pnas.1421504112 25831531 PMC4394283

[2] Ploemacher T, Faber WR, Menke H, Rutten V, Pieters T. Reservoirs and transmission routes of leprosy; A systematic review. PLoS Negl Trop Dis. 2020;14:e0008276. 10.1371/journal.pntd.0008276 32339201 PMC7205316

[3] Avanzi C, Del-Pozo J, Benjak A, Stevenson K, Simpson VR, Busso P, et al. Red squirrels in the British Isles are infected with leprosy bacilli. Science. 2016;354:744–7. 10.1126/science.aah3783 27846605

[4] Colella JP, Bates J, Burneo SF, Camacho MA, Carrion Bonilla C, Constable I, et al. Leveraging natural history biorepositories as a global, decentralized, pathogen surveillance network. PLoS Pathog. 2021;17:e1009583. 10.1371/journal.ppat.1009583 34081744 PMC8174688

[5] Sharma R, Singh P, McCoy RC, Lenz SM, Donovan K, Ochoa MT, et al. Isolation of *Mycobacterium lepromatosis* and development of molecular diagnostic assays to distinguish *Mycobacterium leprae* and *M. lepromatosis.* Clin Infect Dis. 2020;71:e262–9. 10.1093/cid/ciz1121 31732729 PMC8189713

[6] Tió-Coma M, Wijnands T, Pierneef L, Schilling AK, Alam K, Roy JC, et al. Detection of *Mycobacterium leprae* DNA in soil: multiple needles in the haystack. Sci Rep. 2019;9:3165. 10.1038/s41598-019-39746-6 30816338 PMC6395756

[7] Monot M, Honoré N, Garnier T, Zidane N, Sherafi D, Paniz-Mondolfi A, et al. Comparative genomic and phylogeographic analysis of *Mycobacterium leprae.* Nat Genet. 2009;41:1282–9. 10.1038/ng.477 19881526

[8] Sharma R, Singh P, Loughry WJ, Lockhart JM, Inman WB, Duthie MS, et al. Zoonotic leprosy in the southeastern United States. Emerg Infect Dis. 2015;21:2127–34. 10.3201/eid2112.150501 26583204 PMC4672434

[9] Vera-Cabrera L, Ramos-Cavazos CJ, Youssef NA, Pearce CM, Molina-Torres CA, Avalos-Ramirez R, et al. *Mycobacterium leprae* infection in a wild nine-banded armadillo, Nuevo León, Mexico. Emerg Infect Dis. 2022;28:747–9. 10.3201/eid2803.211295 35202538 PMC8888246

[10] Schaub R, Avanzi C, Singh P, Paniz-Mondolfi A, Cardona-Castro N, Legua P, et al. Leprosy transmission in Amazonian countries: current status and future trends. Curr Trop Med Rep. 2020;7:79–91. 10.1007/s40475-020-00206-1

[11] Cardona-Castro N, Escobar-Builes MV, Serrano-Coll H, Adams LB, Lahiri R. *Mycobacterium lepromatosis* as cause of leprosy, Colombia. Emerg Infect Dis. 2022;28:1067–8. 10.3201/eid2805.212015 35450566 PMC9045448

[12] Deps P, Collin SM. *Mycobacterium lepromatosis* as a second agent of Hansen’s disease. Front Microbiol. 2021;12:698588. 10.3389/fmicb.2021.698588 34566911 PMC8461103

[13] Schilling AK, Avanzi C, Ulrich RG, Busso P, Pisanu B, Ferrari N, et al. British red squirrels remain the only known wild rodent host for leprosy bacilli. Front Vet Sci. 2019;6:8. 10.3389/fvets.2019.00008 30775369 PMC6367869

[14] Truman RW, Andrews PK, Robbins NY, Adams LB, Krahenbuhl JL, Gillis TP. Enumeration of *Mycobacterium leprae* using real-time PCR. PLoS Negl Trop Dis. 2008;2:e328. 10.1371/journal.pntd.0000328 18982056 PMC2570796

[15] Schuenemann VJ, Singh P, Mendum TA, Krause-Kyora B, Jäger G, Bos KI, et al. Genome-wide comparison of medieval and modern *Mycobacterium leprae.* Science. 2013;341:179–83. 10.1126/science.1238286 23765279

