# Supplementary material for: Mycobacterium leprae in Armadillo Tissues from Museum Collections, United States
Source: Emerg Infect Dis. 2023 Mar;29(3):622–6. doi: 10.3201/eid2903.221636 (PMC9973711; doi:10.3201/eid2903.221636)
Supplement: Appendix 1 — Additional methods used in study of Mycobacterium leprae in armadillos. [file 22-1636-Techapp-s1.pdf]

Article DOI: https://doi.org/10.3201/eid2903.221636

Mycobacterium leprae in Armadillo Tissues from Museum Collections, United States

Appendix 1

Methods

DNA Extraction

Tissues were processed using an extraction method based on magnetic beads (*1*,*2*). Briefly, the 1 mm^3 tissue sample loaned from 10 U.S. museums (Appendix 1 Table 1) was suspended in a 1.5 mL Eppendorf tube with 290 µL of tissue lysis buffer and 10 µL of proteinase K (20mg/mL), and left at 55°C overnight. After vortexing the samples, the solution was mixed with 300 µL of in-house developed magnetic beads (*2*) and left to rest for 5 minutes in a magnetic tube holder. Each tube was then washed with cold 70% ethanol, dried in a thermal block at 37°C, and resuspended with 200 µL of 1x TE buffer. The cleared solution was extracted and deposited in two different tubes per sample. DNA concentration was measured for each sample using a QuantusTM Fluorometer with the QuantiFluor® ONE dsDNA System (Promega, Madison, WI, U.S.), according to manufacturer instructions.

Molecular Identification

We applied primers specific for detection of *M. leprae* and *M. lepromatosis* as described previously (Appendix 1 Table 2) (*3*–*7*). Primers for detection of *M. leprae* target a segment of 129 base pairs (bp) from the *M. leprae*-specific repetitive element (RLEP). Sensitivity of these primers is high because this region is repeated at least 36 times across the genome (*6*,*7*). For the standard PCR protocol, a final concentration of 25 µL with 3 µL of sample DNA, 2 µL of forward and reverse primers, respectively, at 5 µM initial concentration with 12.5 µL GoTaq® Green Master Mix polymerase (Promega, Madison, WI, USA) at 2x initial concentration, plus molecular-grade water was used.

PCR conditions included denaturation at 95°C for three minutes and 40 cycles of denaturation, annealing, and extension for 30 seconds at 95°C, 40 seconds at 60°C, and 30 seconds at 72°C, respectively. Final extension was at 72°C for 3 minutes. All experiments included a purified sample of *M. leprae* strain Thai53 as a positive control (BEI resource) and molecular-grade water as negative control (*6*,*7*).

The presence of *M. lepromatosis* was screened in all samples using the primers LPM244 suggested by Singh et al. targeting a 244 bp of the *hemN* gene, absent in *M. leprae* (*3*). DNA samples were examined with the same formula and PCR conditions as described above for a final volume of 25 µL. Samples without signs of amplification were reprocessed using primers published half the way of the study that target a multicopy *M. lepromatosis*-specific repetitive element (RLPM (*4*), amplifying a 100 bp segment (Appendix 1 Table 2). PCR conditions were the same as above except for the annealing temperature: 59°C for LPM244 and 65°C for RLPM primers (*3*,*4*,*9*). We processed a total of 89/159 (55.97%) samples with RLPM primers. Although the optimal approach would have been to test all the samples with these primer set, they were unavailable at the start of the experiment and we no longer have molecular material to screen the remaining samples. All examinations for *M. lepromatosis* yielded negative results. Dr. Ramanuj Lahiri from the National Hansen's Disease Program, Louisiana, U.S., kindly provided a positive control for *M. lepromatosis*; molecular-grade water was used as negative control.

Amplified PCR products were inspected in a 2% agarose gel stained with GelRed® Nucleic Acid Stain (Biotium, Fremont, CA, U.S.) and a 50 bp molecular ladder (Fisher Scientific, Hanover Park, IL, U.S.) using an ultraviolet light trans illuminator (Appendix 1 Figures 1–3). We processed all tissues twice and considered positive any of those with readable bands on the expected fragment sizes on the electrophoresis gel. Amplicons were sequenced by Genewiz and Functional Biosciences. High quality sequences were either aligned or directly compared with published *M. leprae* and *M. lepromatosis* sequences via the basic local alignment search tool (BLAST) using default parameters from Geneious Prime® 2022.0.1.

PCR Subtypification

Subtypification via PCR-based sequencing was performed for all positive samples as follows. We first used a set of primers to differentiate *M. leprae* types between either 1–2 or 3–4 (Appendix 1 Table 3) (*10*). Then, we used a previously described variant to identify subtype 3I, considering that this is the genotype expected to be circulating in North America (*9,11*). PCR conditions were implemented as mentioned above, with annealing temperatures calibrated for each primer (Appendix 1 Table 3). Amplicons were sequenced as described above.

Quantitative PCR and Whole-Genome Sequencing

Quantitative real-time PCR (qPCR) was performed on all samples for which genotyping was successful to obtain a proxy of *M. leprae* DNA quantity as an assessment for subsequent genome sequencing steps (*8*). Briefly, the repetitive element RLEP was quantified using TaqMan® PCR amplification as described previously, with minor modifications (*12*). A total of 3 µL of each purified DNA sample, or positive (i.e., DNA from Thai-53, NR-19352) or the control (i.e., nuclease-free water), was added to a total PCR reaction volume of 20 µL, containing 10 µL of SsoAdvanced Universal Probes Supermix (Biorad, CA, U.S.), 900 nM of each forward (RLEPq-F) and reverse (RLEPq-R) primer, and 250 nM of the hydrolysis probe (RLEPq-P) (Appendix 1 Table 2). Reaction mixtures were prepared in duplicate, and amplification started with an initial denaturation step of 10 minutes at 95°C and 1 minute at 60°C, using the CFX96 real-time PCR system (BioRad, CA, USA). Data analysis was performed with the CFX Maestro Software (BioRad, CA, USA), and the mean cycle threshold (Ct) was calculated for each sample.

All samples with Ct<26 were prepared for whole genome sequencing (WGS). Briefly, around 100 ng of extracted DNA was fragmented to 300 bp using a Covaris M220 focused ultrasonicator and the MICROtube-130, as recommended by the manufacturer (Covaris, MA, U.S.), followed by a 1.8x AMPure bead clean-up. DNA libraries were prepared using the Kapa HyperPrep Kits, the KAPA universal adaptor and the KAPA UDI primer mix for indexing, and the target enriched capture using the KAPA HyperExplore protocol (kit KAPA HyperExplore Probe protocol, hybridization for 24 hours; Roche, Switterland). The quality of the DNA library fragment was assessed using

the Screen Tape D1000 on an Agilent TapeStation 4100 instrument (Agilent, CA, U.S.), and the library was quantified via Qubit (ThermoFisher, MA, U.S.). The libraries were multiplexed and sequenced using single-end reads on Illumina NextSeq 500 instrument. Raw reads were processed as described elsewhere (*13*). A maximum parsimony (MP) tree was constructed in MEGA version 11 (*14*), with the two new genomes from this study and the genomes from Vera-Cabrera et al. (*9*), using 500 bootstrap replicates and *M. lepromatosis* as an outgroup.

Supplementary References

1. Rohland N, Reich D. Cost-effective, high-throughput DNA sequencing libraries for multiplexed target capture. Genome Res. 2012;22:939–46. PubMed https://doi.org/10.1101/gr.128124.111

2. Oaks J. SPRI Bead DNA Extraction Protocol. 2020 [cited 2022 Oct 17]. https://github.com/phyletica/lab-protocols/blob/master/extraction-spri.md

3. Singh P, Benjak A, Schuenemann VJ, Herbig A, Avanzi C, Busso P, et al. Insight into the evolution and origin of leprosy bacilli from the genome sequence of *Mycobacterium lepromatosis*. Proc Natl Acad Sci U S A. 2015;112:4459–64. PubMed https://doi.org/10.1073/pnas.1421504112

4. Sharma R, Singh P, McCoy RC, Lenz SM, Donovan K, Ochoa MT, et al. Isolation of *Mycobacterium lepromatosis* and development of molecular diagnostic assays to distinguish *Mycobacterium leprae* and *M. lepromatosis*. Clin Infect Dis. 2020;71:e262–9. PubMed https://doi.org/10.1093/cid/ciz1121

5. Braet S, Vandelannoote K, Meehan CJ, Brum Fontes AN, Hasker E, Rosa PS, et al. The repetitive element RLEP is a highly specific target for detection of *Mycobacterium leprae*. J Clin Microbiol. 2018;56:e01924–17. PubMed https://doi.org/10.1128/JCM.01924-17

6. Donoghue HD, Holton J, Spigelman M. PCR primers that can detect low levels of *Mycobacterium leprae* DNA. J Med Microbiol. 2001;50:177–82. PubMed https://doi.org/10.1099/0022-1317-50-2-177

7. Tió-Coma M, Wijnands T, Pierneef L, Schilling AK, Alam K, Roy JC, et al. Detection of *Mycobacterium leprae* DNA in soil: multiple needles in the haystack. Sci Rep. 2019;9:3165. PubMed https://doi.org/10.1038/s41598-019-39746-6

8. Avanzi C, Lécorché E, Rakotomalala FA, Benjak A, Rapelanoro Rabenja F, Ramarozatovo LS, et al. Population genomics of *Mycobacterium leprae* reveals a new genotype in Madagascar and the Comoros. Front Microbiol. 2020;11:711. PubMed https://doi.org/10.3389/fmicb.2020.00711

9. Vera-Cabrera L, Ramos-Cavazos CJ, Youssef NA, Pearce CM, Molina-Torres CA, Avalos-Ramirez R, et al. *Mycobacterium leprae* infection in a wild nine-banded armadillo, Nuevo León, Mexico. Emerg Infect Dis. 2022;28:747–9. PubMed https://doi.org/10.3201/eid2803.211295

10. Monot M, Honoré N, Garnier T, Zidane N, Sherafi D, Paniz-Mondolfi A, et al. Comparative genomic and phylogeographic analysis of *Mycobacterium leprae.* Nat Genet. 2009;41:1282–9. PubMed https://doi.org/10.1038/ng.477

11. Truman RW, Singh P, Sharma R, Busso P, Rougemont J, Paniz-Mondolfi A, et al. Probable zoonotic leprosy in the southern United States. N Engl J Med. 2011;364:1626–33.

12. Truman RW, Andrews PK, Robbins NY, Adams LB, Krahenbuhl JL, Gillis TP. Enumeration of *Mycobacterium leprae* using real-time PCR. PLoS Negl Trop Dis. 2008;2:e328. PubMed https://doi.org/10.1371/journal.pntd.0000328

13. Benjak A, Avanzi C, Singh P, Loiseau C, Girma S, Busso P, et al. Phylogenomics and antimicrobial resistance of the leprosy bacillus *Mycobacterium leprae.* Nat Commun. 2018;9:352. PubMed https://doi.org/10.1038/s41467-017-02576-z

14. Tamura K, Stecher G, Kumar S. MEGA11: Molecular Evolutionary Genetic Analysis version 11. Mol Biol Evol. 2021;38:3022–7. PubMed https://doi.org/10.1093/molbev/msab120

Appendix 1 Table 1. Museums from the United States with available armadillo tissues*

Museum	No. samples	Website
Museum of Texas Tech University. Natural Science Research Laboratory	46	https://www.depts.ttu.edu/nsrl/collections/search-database.php
Peabody Museum of Natural History	33	https://peabody.yale.edu/explore/collections
Angelo State Natural History Collections	27	https://www.angelo.edu/departments/biology/angelo-state-natural-history-collection/
Sam Noble Oklahoma Museum of Natural History	17	https://samnoblemuseum.ou.edu/
Museum of Southwestern Biology	12	https://msb.unm.edu/divisions/mammals/index.html
University of Alaska Museum of the North	9	https://www.uaf.edu/museum/
Louisiana Museum of Natural History	7	https://www.lsu.edu/mns/collections/mammalogy.php
Museum of Vertebral Zoology	5	https://mvz.berkeley.edu/mvzmamm/
Florida Museum of National History	2	https://www.floridamuseum.ufl.edu/collections/
Field Museum of Natural History	1	https://www.fieldmuseum.org/science/research
Total	159	

*Museums ordered according to the number of total individual armadillos contributed to this study.

Appendix 1 Table 2. Primers used to identify *Mycobacterium leprae* and *M. lepromatosis**

Species	Primer	Sequence (5′→3′)	Annealing temperature, °C	Amplicon size, bp	Source
Mycobacterium leprae	LP1 (F)	TGCATGTCATGGCCTTGAGG	60	129	(*6*,*7*)
	LP2 (R)	CACCGATACCAGCGGCAGAA			
M. lepromatosis	LPM244-F	GTTCCTCCACCGACAAACAC	59	244	(*3*)
	LPM244-R	TTCGTGAGGTACCGGTGAAA			
M. lepromatosis	RLPM-F	TTGGTGATCGGGGTCGGCTGGA	65	100	(*4*)
	RLPM-R	CCCCACCGGACACCACCAACC			
M. leprae (qPCR)	RLEPq-F	GCAGTATCGTGTTAGTGAA	60	-	(*8*)
	RLEPq-R	CGCTAGAAGGTTGCCGTATG	60	-	
	RLEPq-P	FAM-TCGATGATCCGGCCGTCGGCG-QSY	60	-	

*Each primer was calibrated locally to obtain the most adequate annealing temperature using the *Mycobacterium leprae*/*M. lepromatosis* positive control. bp, base pairs.

Appendix 1 Table 3. Primers used for *Mycobacterium leprae* PCR subtyping*

Primer name	Type/ Subtype	Gene targeted	Sequence (5′→3′)	Nucleic acid change	Annealing temperature, °C	Amplicon size, bp	Source
SNP-73-F	Type 1–2 or 3–4	*dnaA*	CCCGAAATTTACGAGAACCA	A73G	58	200	(*10*)
SNP-73-R			AATCCCTCGATGATGGTGAG				
gyrA (3I)-F	Subtype 3I	*gyrA*	TAAGTCAGCACGGTCAGTCG	C7614T	58	213	Adapted from Truman et al. 2011 (*11*)
gyrA (3I)-R			TCCCAAATAGCAACCTCACC				

*Each primer was calibrated locally to obtain the most adequate annealing temperature using the *M. leprae* positive control. bp, base pairs.

Appendix 1 Table 4. Whole-genome sequencing results for the samples available in this study (n = 2)

Parameters	Sample 109 (SAMN31421191)	Sample 209 (SAMN31421192)
Total number of reads	9,024,266	10,029,143
Percentage of reads mapping to the reference genome TN (AL450380)	88.16%	88.16%
Coverage (no duplicate)	18.2x	4.9x

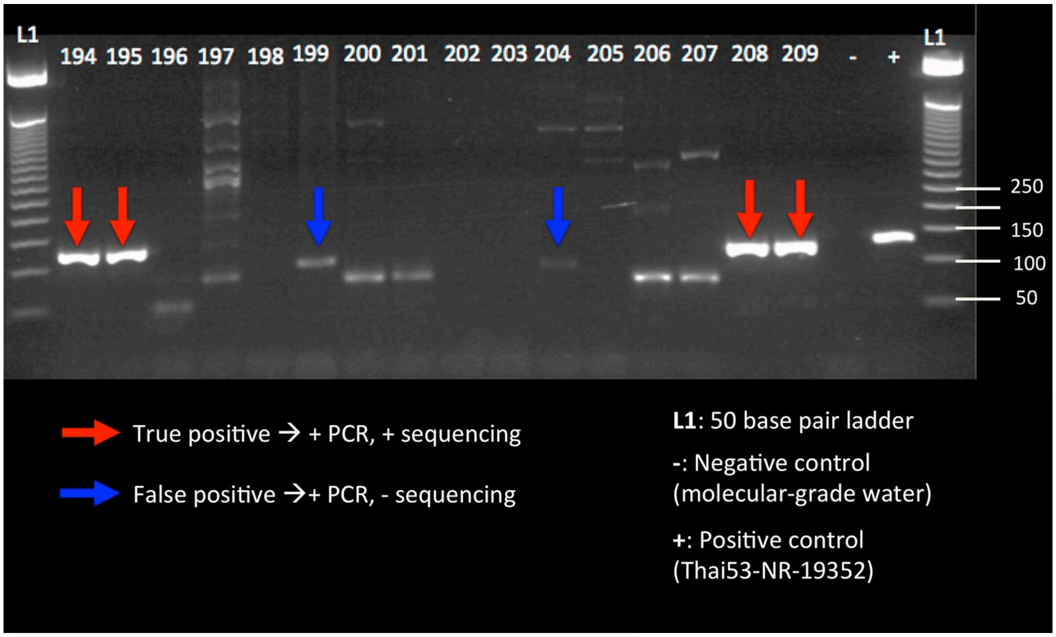

Appendix 1 Figure 1. Example of gel electrophoresis for the identification of *Mycobacterium leprae*. All PCR positives were sequenced to confirm their status as true positives or true negatives. Positive control Thai53-NR-19352.

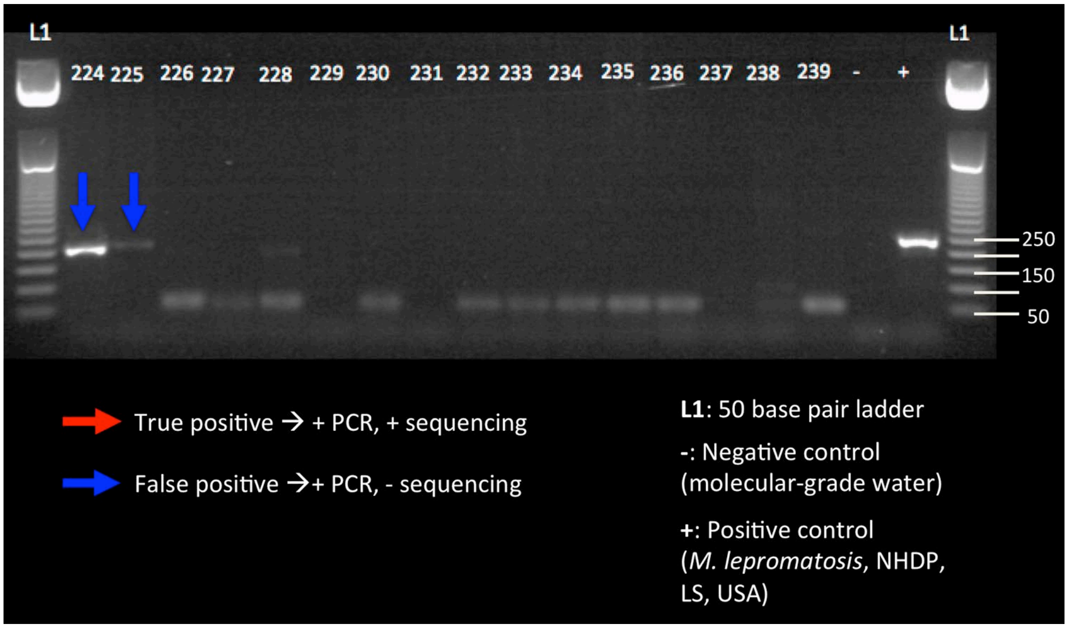

Appendix 1 Figure 2. Example of gel electrophoresis for the identification of *Mycobacterium lepromatosis* with primers LPM244. All PCR positives were sequenced to confirm their status as true positives or true negatives. *Mycobacterium lepromatosis* was donated by Dr. Ramanuj Lahiri from the National Hansen's Disease Program, Louisiana, US. All samples were negative across the examinations.

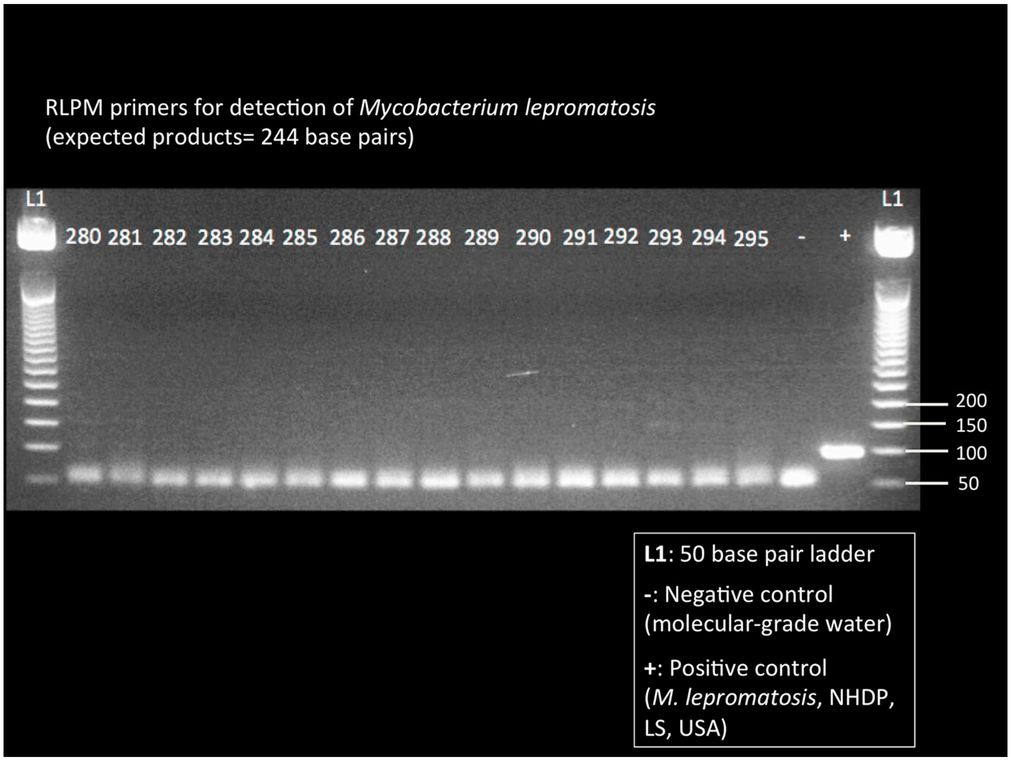

Appendix 1 Figure 3. Example of gel electrophoresis for the identification of *Mycobacterium lepromatosis* with primers RLPM. All PCR positives were sequenced to confirm their status as true positives or true negatives. *Mycobacterium lepromatosis* was donated by Dr. Ramanuj Lahiri from the National Hansen's Disease Program, Louisiana, US. All samples were negative across the examinations.

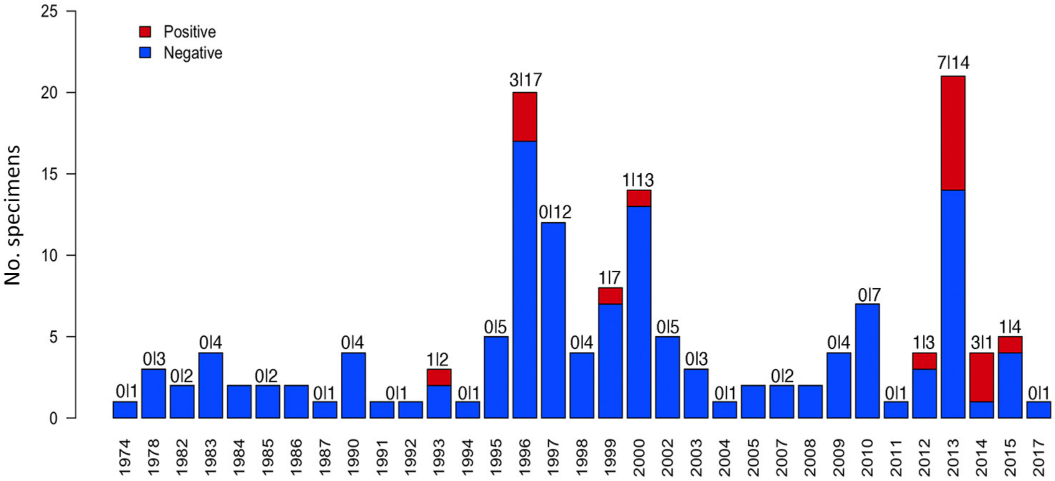

Appendix 1 Figure 4. Armadillo collections from our sample included tissues from 1974 through 2017. Numbers above the bars represent the number of samples that tested negative and positive for *Mycobacterium leprae* for each year.
